# Severe T-System Remodeling in Pediatric Viral Myocarditis

**DOI:** 10.3389/fcvm.2020.624776

**Published:** 2021-01-18

**Authors:** Dominik J. Fiegle, Martin Schöber, Sven Dittrich, Robert Cesnjevar, Karin Klingel, Tilmann Volk, Muhannad Alkassar, Thomas Seidel

**Affiliations:** ^1^Institute of Cellular and Molecular Physiology, Friedrich-Alexander-Universität Erlangen-Nürnberg, Erlangen, Germany; ^2^Department of Pediatric Cardiology, Friedrich-Alexander-Universität Erlangen-Nürnberg, Erlangen, Germany; ^3^Department of Pediatric Cardiac Surgery, University Hospital Erlangen, Erlangen, Germany; ^4^Cardiopathology, University Hospital Tuebingen, Tübingen, Germany; ^5^Muscle Research Center Erlangen (MURCE), Friedrich-Alexander-Universität Erlangen-Nürnberg, Erlangen, Germany

**Keywords:** myocarditis, transverse tubular system, excitation-contracting coupling, heart failiure, remodeling, confocal micoscopy, ryanodine receptor (RyR)

## Abstract

Chronic heart failure (HF) in adults causes remodeling of the cardiomyocyte transverse tubular system (t-system), which contributes to disease progression by impairing excitation-contraction (EC) coupling. However, it is unknown if t-system remodeling occurs in pediatric heart failure. This study investigated the t-system in pediatric viral myocarditis. The t-system and integrity of EC coupling junctions (co-localization of L-type Ca^2+^ channels with ryanodine receptors and junctophilin-2) were analyzed by 3D confocal microscopy in left-ventricular (LV) samples from 5 children with myocarditis (age 14 ± 3 months), undergoing ventricular assist device (VAD) implantation, and 5 children with atrioventricular septum defect (AVSD, age 17 ± 3 months), undergoing corrective surgery. LV ejection fraction (EF) was 58.4 ± 2.3% in AVSD and 12.2 ± 2.4% in acute myocarditis. Cardiomyocytes from myocarditis samples showed increased t-tubule distance (1.27 ± 0.05 μm, *n* = 34 cells) and dilation of t-tubules (volume-length ratio: 0.64 ± 0.02 μm^2^) when compared with AVSD (0.90 ± 0.02 μm, *p* < 0.001; 0.52 ± 0.02 μm^2^, *n* = 61, *p* < 0.01). Intriguingly, 4 out of 5 myocarditis samples exhibited sheet-like t-tubules (t-sheets), a characteristic feature of adult chronic heart failure. The fraction of extracellular matrix was slightly higher in myocarditis (26.6 ± 1.4%) than in AVSD samples (24.4 ± 0.8%, *p* < 0.05). In one case of myocarditis, a second biopsy was taken and analyzed at VAD explantation after extensive cardiac recovery (EF from 7 to 56%) and clinical remission. When compared with pre-VAD, t-tubule distance and density were unchanged, as well as volume-length ratio (0.67 ± 0.04 μm^2^ vs. 0.72 ± 0.05 μm^2^, *p* = 0.5), reflecting extant t-sheets. However, junctophilin-2 cluster density was considerably higher (0.12 ± 0.02 μm^−3^ vs. 0.05 ± 0.01 μm^−3^, *n* = 9/10, *p* < 0.001), approaching values of AVSD (0.13 ± 0.05 μm^−3^, *n* = 56), and the measure of intact EC coupling junctions showed a distinct increase (20.2 ± 5.0% vs. 6.8 ± 2.2%, *p* < 0.001). Severe t-system loss and remodeling to t-sheets can occur in acute HF in young children, resembling the structural changes of chronically failing adult hearts. T-system remodeling might contribute to cardiac dysfunction in viral myocarditis. Although t-system recovery remains elusive, recovery of EC coupling junctions may be possible and deserves further investigation.

## Introduction

The cardiomyocyte transverse tubular system (t-system), a dense network of membrane invaginations (t-tubules), is essential for efficient Ca^2+^ cycling and excitation-contraction (EC) coupling. T-tubules guarantee the formation and maintenance of a large number of close junctions between L-type Ca^2+^ channels (LTCC) and ryanodine receptors (RyR), which is facilitated by junctophilin-2, a protein spanning the distance between the plasma and SR membranes and interacting with both LTCCs and RyRs (1–4). Upon electrical activation, Ca^2+^ enters the cell through LTCC, binds to adjacent RyRs and elicits additional Ca^2+^ release from the sarcoplasmic reticulum. The resulting increase in cytosolic Ca^2+^ concentration triggers cardiomyocyte contraction.

With a complex 3D structure and diameters ranging from 0.1 to 0.5 μm, t-tubules can be visualized best by 3D confocal microscopy and related imaging techniques (5). In conjunction with computational image analysis, pathological alterations of the t-system can be quantified in cardiac disease. Remodeling and loss of the t-system are common features of chronic heart failure in adults, impairing EC coupling, contributing to disease progression and limiting recovery (6–11). It was reported that a sheet-like structure of t-tubules, resulting from one-dimensional dilation along the myocyte long axis, is a typical feature of human heart failure (11), and similar structures were observed in animal models of cardiac disease (12, 13). However, as most studies investigated either animal or adult human hearts, little is known about the t-system in young children. In particular, it is unknown if t-system remodeling does also occur in acute heart failure in children.

A common cause of acute pediatric heart failure is fulminant viral myocarditis (14), but the mechanisms underlying contractile dysfunction and recovery in myocarditis remain elusive (15). Cell death (16), metabolic alterations (17), and immune response mechanisms (18) have been described, but data on structures of cardiac EC coupling, such as the t-system or EC coupling junctions, are rare (19). Thus, it is unclear if they are affected by acute myocarditis.

This study describes and displays severe t-system loss and remodeling to t-sheets in pediatric patients with acute viral myocarditis and explores a case of partial structural recovery of EC coupling junctions during mechanical circulatory support.

## Methods

### Human Cardiac Specimens

Left-ventricular myocardial samples and endomyocardial biopsies were collected during implantation and explantation of ventricular assist devices from pediatric hearts with viral myocarditis (age 17 ± 3, *n* = 5), and during atrioventricular septal defect (AVSD) surgeries in children of comparable age (14 ± 3 months, *n* = 5). See Table 1 for details. The study was approved by the local institutional review boards and followed the declaration of Helsinki principles. Legal guardians of all patients gave their written informed consent.

**Table 1:** Patient data.

**No**	**Region**	**Sex**	**Age [months]**	**LVEF [%]**	**Diagnosis**
**AVSD**					
1	LVOT	M	12.9	62	AVSD
2	LVOT	M	17.3	58	AVSD and hypertrophic cardiomyopathy
3	LVOT	F	17.7	64	VSD
4	LVOT	M	27.5	55	AVSD
5	LVOT	M	9.1	53	VSD
**Myocarditis**					
1	LV	F	21.5	7	Acute myocarditis (adenovirus and HHV6)
2	LV	F	6.3	13	Acute myocarditis (adenovirus)
3	LV	F	16.4	11	Acute myocarditis (HHV6/7)
4	LV	F	15.2	10	Acute myocarditis (PVB19 and HHV6)
5	LV	M	12.4	20	Acute myocarditis (PVB19)

*Clinical data comprising myocardial biopsy region, sex (m, male; f, female), age at time of surgery, left ventricular ejection fraction (LVEF) at time of surgery and the diagnosis. The order is consistent with Figure 4. AVSD, atrioventricular septal defect; VSD, ventricular septal defect; HHV, Human herpes virus (subtype 6 or 7); PVB19, Parvovirus B19; LVOT, left-ventricular outflow tract*.

### Echocardiography

Echocardiographic examinations of myocarditis patients were carried out before VAD implantation. In one patient, additional echocardiographic examinations were performed 1 month after VAD implantation and directly before VAD explantation. In AVSD patients, echocardiography was performed few days before or after surgery to assess ventricular function. In all patients, left ventricular ejection fraction (LVEF) was calculated according to Simpson, from end-diastolic and end-systolic optical sections in 2- and 4-chamber view. In some patients, regional wall-movements, and global longitudinal systolic strain (GLS) were analyzed with speckle tracking.

### Histopathological Diagnostics

Formalin-fixed, paraffin-embedded heart tissue probes were examined by histopathological (Dallas criteria) and immunohistological methods for the identification of inflammatory infiltrates. In some samples, Masson's trichrome stain was used to visualize collagen fibers. Samples for virus diagnostics were snap-frozen in liquid nitrogen and stored at −80°C. After RNA-DNA extraction, genome amplification of cardiotropic viruses was performed by realtime (RT) PCR or nested RT-PCR, according to the ESC recommendation (20).

### Tissue Preparation

Myocardial biopsies were either used instantly for cardiomyocyte isolation, snap-frozen in liquid nitrogen or fixed with PFA and embedded in paraffin, using standard methods. Frozen tissue was embedded in sectioning compound (TissueTek O.C.T, 4583) and cut at −20°C with a cryotome into 60 μm thick sections. Sections were immediately fixed in paraformaldehyde (PFA, 2 % in PBS) for 10 min and then used for immunostaining. Paraffin-embedded samples were processed to 30 μm thick sections on a microtome and rehydrated in a xylol/ethanol dilution series on microscope slides before staining.

### Cardiomyocyte Isolation

Cardiomyocytes were isolated as described previously (21). In brief, tissue specimens were embedded in low melting-point agarose (Roth, 4 % w/v) and cut into 300 μm thick sections with a vibratome (Leica VT1200 S), while being submerged in cutting solution [containing in mM: 138 NaCl, 0.33 NaH_2_PO4, 5.4 KCl, 2 MgCl_2_, 0.5 CaCl_2_, 10 HEPES, 10 glucose, 30 butanedione monoxime (BDM), pH 7.4]. The agarose was then removed, and the slices were rinsed twice with isolation solution [containing in mM: 10 beta-hydroxybutyrate, 30 BDM, 10 glucose, 70 glutamic acid, 20 KCl, 10 KH_2_PO_4_, 10 MgCl_2_, 20 taurin and 2% bovine serum albumin (BSA), pH 7.4], transferred into a petri dish and isolated by enzymatic digestion at 37°C with constant agitation on a rocker. First, 0.5 mg/ml proteinase [type XXIV, (~3.5–7 U/ml), Sigma, P8038] was applied for 12 min. After two washing steps 4 mg/ml collagenase (type I, 330 U/mg, Merck, CAS 9001-12-1) and 5 μM CaCl_2_ were applied for 30 min. Next, the Ca^2+^ concentration in the solution was elevated in several steps and BDM was washed out gradually. Cardiomyocytes were immediately used for subsequent experiments.

### Fluorescent Staining

Living isolated cardiomyocytes were stained in a modified Tyrode's Solution (containing in mM: 130 NaCl, 0.4 NaH_2_PO_4_, 5.8 NaHCO_3_, 5.4 KCl, 0.5 MgCl_2_, 25 HEPES, 22 glucose, 2 CaCl_2_, 30 BDM) with 8 μM of the lipophilic membrane dye Di8-ANEPPS (Di8, Enzo LifeSciences, ENZ-52204). After a minimum of 20 min incubation at room temperature, the cells were imaged by confocal microscopy.

Excitation-contraction (EC) coupling proteins junctophilin-2 (JPH2), L-type Ca^2+^ channel (LTCC), and ryanodine receptor subtype 2 (RyR) were stained by immunofluorescence in PFA-fixed, isolated cardiomyocytes or myocardial tissue slices. Primary antibodies against JPH2 (Thermo Fisher, 40-5300), LTCC (Alomone, AGP-001), and RyR (Thermo Fisher, MA3 916) were diluted 1:200 in staining solution (phosphate buffered saline, PBS, supplemented with 1% BSA, 5% normal goat serum, 0.25% TritonX-100) and incubated for a minimum of 16h in the dark at 4°C. After washing with PBS the specimen were incubated with the nuclear stain, 4′,6- diamidino-2-phenylindole (DAPI, Roth, 6335.1, 1 μM) and fluorophore-conjugated secondary antibodies, goat anti-rabbit Pacific Orange (Thermo Fisher, P31584), goat anti-guinea pig AF647 (Thermo Fisher, A21450), goat anti-mouse AF488 (Thermo Fisher, A21121), at 1:200 dilution for 4 h at room temperature. After two final washing steps, the stained cardiomyocytes were imaged by confocal microscopy in PBS. Tissue sections were additionally stained for 3 h at room temperature with wheat germ agglutinin (WGA-CF555, Biotium, 29076, 40 μg/ml in PBS). The sections were then washed twice with PBS, embedded in Fluoromount G (Invitrogen, 00-4958-02) on a microscope slide, covered with a coverslip and allowed to dry for a minimum of 24 h before confocal microscopy.

### Confocal Imaging

For confocal imaging, cardiomyocyte suspension was transferred to a dish with coverslip bottom (thickness: 0.15 mm) and mounted on the stage of a Leica LSM780 inverted confocal microscope. Microscope slides with embedded tissue sections were directly mounted on the stage. Confocal image stacks of 1,280 × 384 × 26 to 1,536 × 512 × 52 pixels, for single cells, and 1,280 × 1,280 × 125 pixels, for tissue sections, with a voxel size of 0.1 × 0.1 × 0.2 μm^3^ were acquired using a 63x oil immersion lens (Zeiss Plan Apochromat, NA 1.4). The pixel dwell time was set to 1.26 μs and signal attenuation in depth was corrected by gradual laser power increase (22). Di8-ANEPPS was excited at 488 nm with an argon laser and emission was recorded at 507-690 nm. Multilabel-stained cells, were excited sequentially in three separate tracks with [1] 405 nm, [2] 488 and 633 nm, and [3] 561 nm. Emission was detected at 418–478 nm, 490–515 nm and [1] 531–704 nm, [2] 496–576 nm, and [3] 638-735 nm and 560-665 nm.

### Image Processing and Analysis

Confocal image stacks were noise-filtered, deconvolved by application of the Richardson-Lucy algorithm, and corrected for depth-dependent signal attenuation (22). Multilabel fluorescence images of EC coupling proteins were corrected for spill-over between AF488 and Pacific Orange by linear unmixing according to published methods (23, 24). Morphological watershed segments were generated and manually refined to obtain cell masks of isolated cardiomyocytes and for individual cells from whole tissue sections, in the case of AVSD samples (22, 25). Cardiomyocyte masks were morphologically closed and used for further analysis of intracellular components. Histogram-based thresholds were applied to separate signal from background. T-system measures, i.e. intracellular distance of each voxel to the closest t-tubule (TT distance), t-system volume normalized to cell volume (TT density), and volume-to-length ratio of t-tubules (TT shape) were calculated as described (11, 24). EC coupling protein clusters (RyR, LTCC, JPH2) were detected by connected-component analysis. Cluster densities were calculated as the number of clusters per cell volume (clusters per μm^3^). Co-localization was defined as overlapping fraction between clusters. The percentage of LTCC clusters co-localized with both RyR and JPH2 clusters was used as a measure of intact EC coupling junctions. Three-dimensional representations were reconstructed from segmented images, using ParaView (v5.8). The amount of extracellular matrix as an indicator of fibrosis was measured by calculating the volume fraction of WGA-stained image regions after application of a histogram-based threshold (image mode + standard deviation) (13, 26).

### Statistics

Data in the text and figures are reported as mean ± standard error. One-way analysis of variance (ANOVA) was used to test if the samples stemmed from different populations. Linear mixed-effects models were applied for AVSD and myocarditis samples to account for repeated measures (cells) per sample and for possible variance resulting from sample preparation or cell isolation (27, 28). The intercept of each sample was included as a random effect according to the following model: Y ~ X + (1 | G), for dependend variable Y (response), predicting variable X (fixed effect) and grouping variable G with a possible random effect on the intercept (1). As grouping variable, the sample ID was used. The two-tailed unequal variances t-test was applied to test for significant differences between pre- and post-VAD groups, with the level of significance set to p < 0.05. Holm-Bonferroni multiple comparison correction was applied where required.

## Results

### Viral Myocarditis as a Cause of Acute Heart Failure in Young Children

We studied left-ventricular samples from 5 children with viral myocarditis undergoing VAD implantation and 5 children with atrioventricular septum defect undergoing corrective surgery. AVSD hearts served as control, because cardiac samples from age-matched healthy subjects were not available. An overview of the patients is presented in Table 1. Mean LVEF of myocarditis hearts at time of VAD implantation was 12.2 ± 2.8%, whereas AVSD hearts exhibited normal systolic function (EF = 58.4 ± 2.3%). Except for one patient with hypertrophic cardiomyopathy, left-ventricular dimensions were normal in the AVSD group. Myocarditis and AVSD patients were of similar age (17 ± 3 months and 14 ± 3 months, respectively).

### T-System Loss and Remodeling in Acute Myocarditis

**Figure 1:**
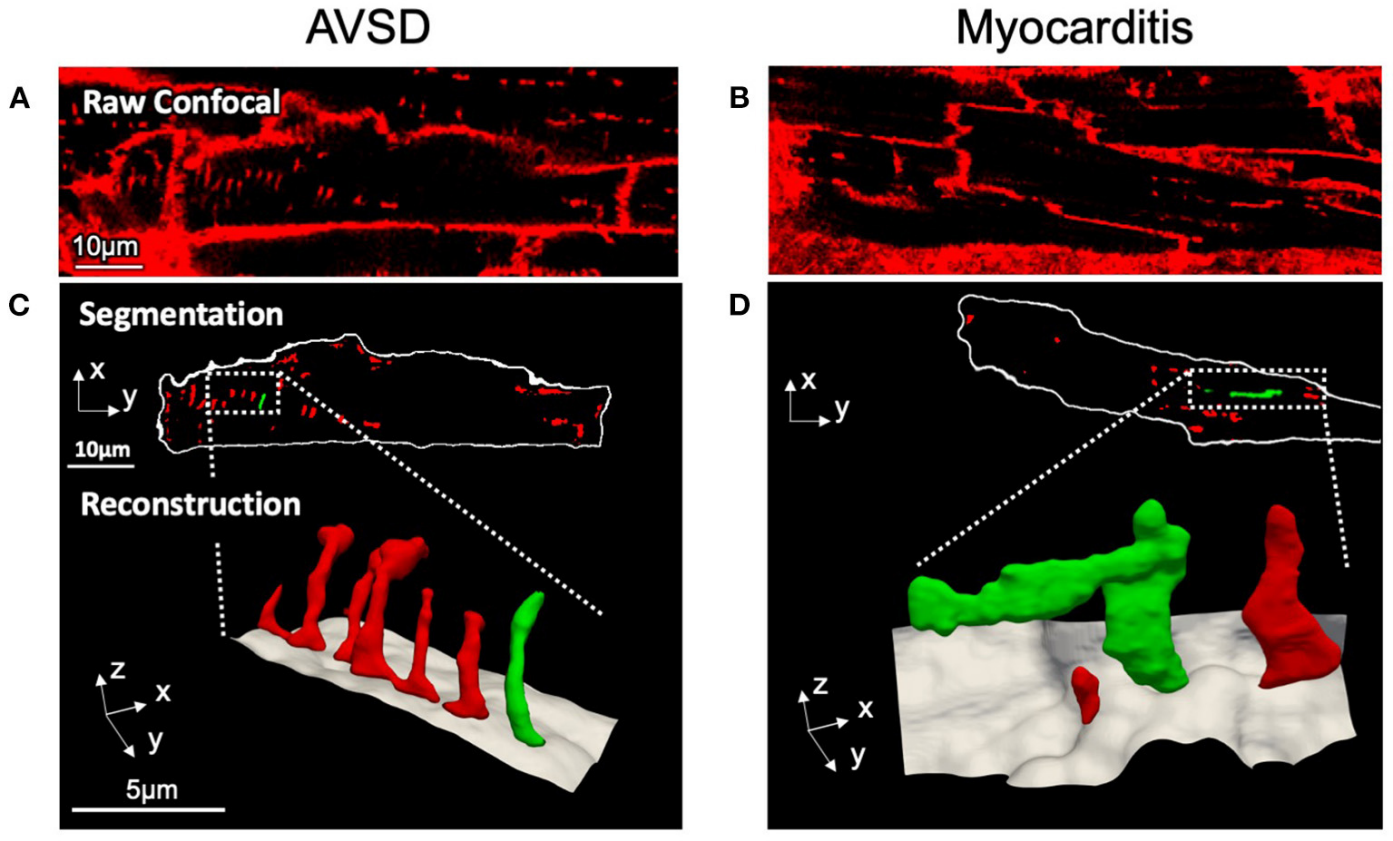
Confocal microscopic images and 3D t-system reconstruction of LV tissue samples from pediatric patients with AVSD (left) or fulminant myocarditis (right). **(A,B)** Raw confocal images of myocardial tissue sections stained with WGA. **(C,D)** The surface membrane (white) and t-system (red, green) were discerned by image segmentation. Reconstructions represent three-dimensional views of the highlighted areas, with surface membranes (gray) and the t-system. Scale bars in **(A,C)** also apply to **(B,D)**, respectively.

To investigate if t-system structure may be affected in viral myocarditis, cardiomyocytes from AVSD and myocarditis patients were imaged by 3D confocal microscopy. Figure 1 shows exemplary cells from the two groups. The AVSD cell exhibited a regular t-system with densely arranged tubular elements. In striking contrast, the myocarditis cardiomyocyte exhibited not only a lower number of t-tubules, but also dilation of individual t-system components to sheet-like structures (t-sheets), which has been described in chronic heart failure of adult patients and been linked to impaired excitation-contraction coupling and recovery (11).

**Figure 2:**
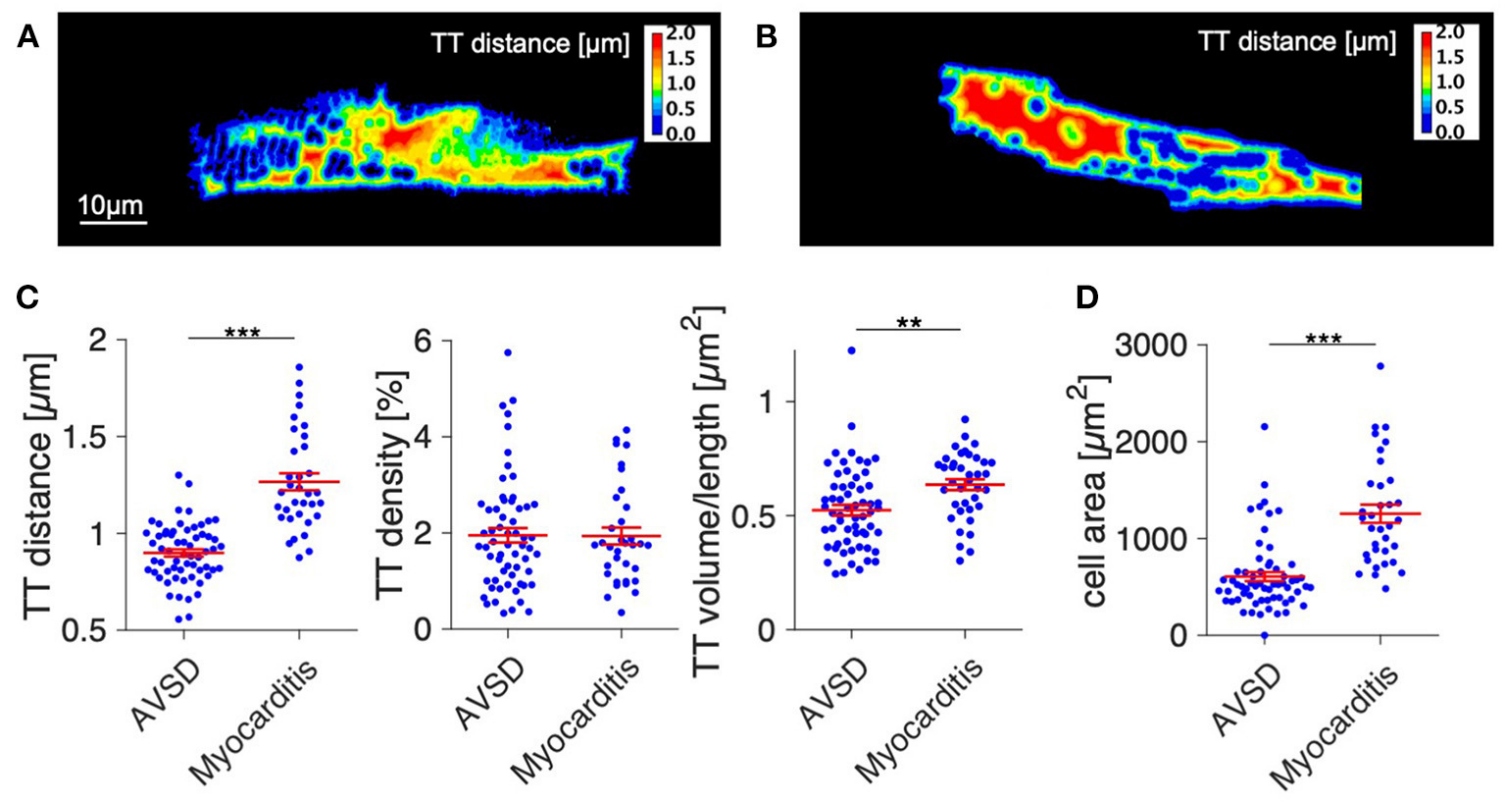
Quantification of t-system remodeling and cell size in AVSD and acute viral myocarditis. **(A,B)** Distance maps of the cells shown in Figure 1, with color-coded intracellular distance to the nearest t-tubule in μm (TT distance). **(A)** Example cell from AVSD, **(B)** Example cell from myocarditis. **(C)** Quantification of the intracellular t-tubule distance (TT distance), volume density (TT density), and shape (TT volume/length). **(D)** Cell area. Number of analyzed cells/patients: 61/5 (AVSD), 34/5 (myocarditis). **p < 0.01, ***p < 0.001, linear mixed-effects model with patient group (AVSD or myocarditis) as predicting variable and intercept as random effect by patient (i.e., sample). Scale bar in **(A)** also applies to **(B)**.

**Figure 3:**
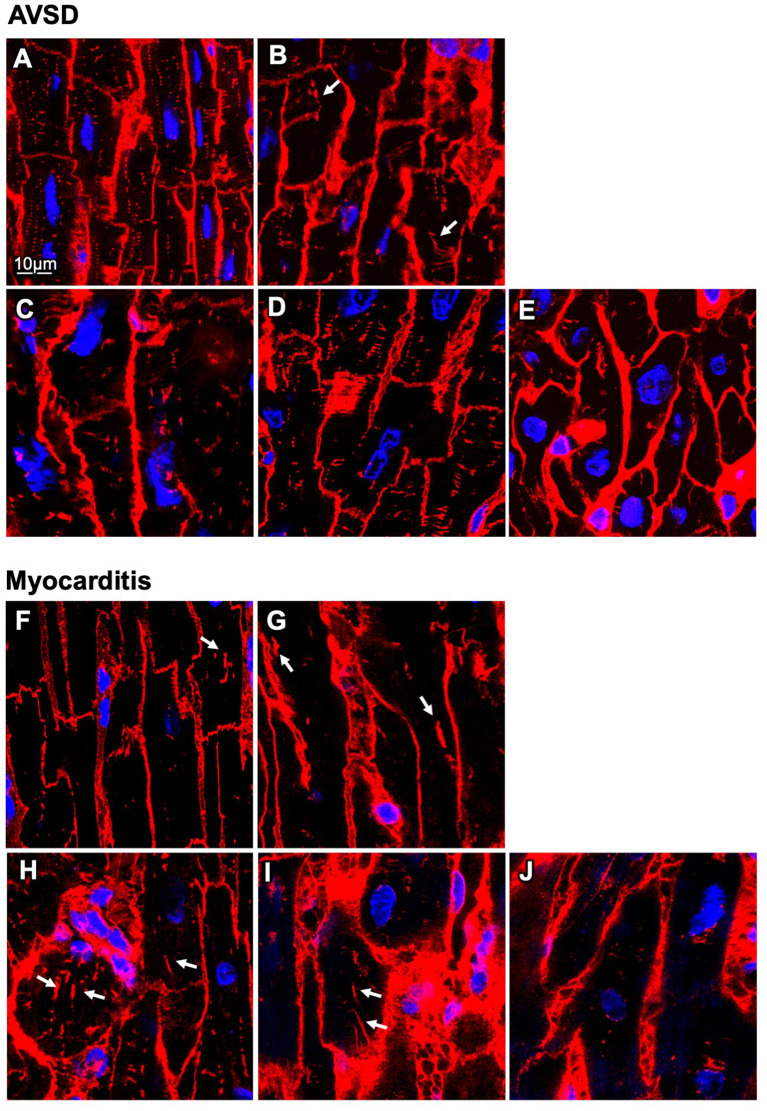
Confocal microscopic images of myocardial samples from AVSD and myocarditis patients. **(A–E)** Tissues obtained from five pediatric patients with atrioventricular septal defect (AVSD), stained with WGA (red), and DAPI (blue) **(F–J)** Tissues obtained from five pediatric patients with myocarditis. Enlarged t-system components (t-sheets) are indicated with white arrows. The tissue in **(F)** was retrieved at time of VAD explantation, **(G–J)** at time of implantation. Note the low t-system density in **(F–J)** when compared with **(A–E)** and that t-sheets appear as longitudinal components in the xy view. Scale bar in **(A)** also applies to **(B–J)**.

**Figure 4:**
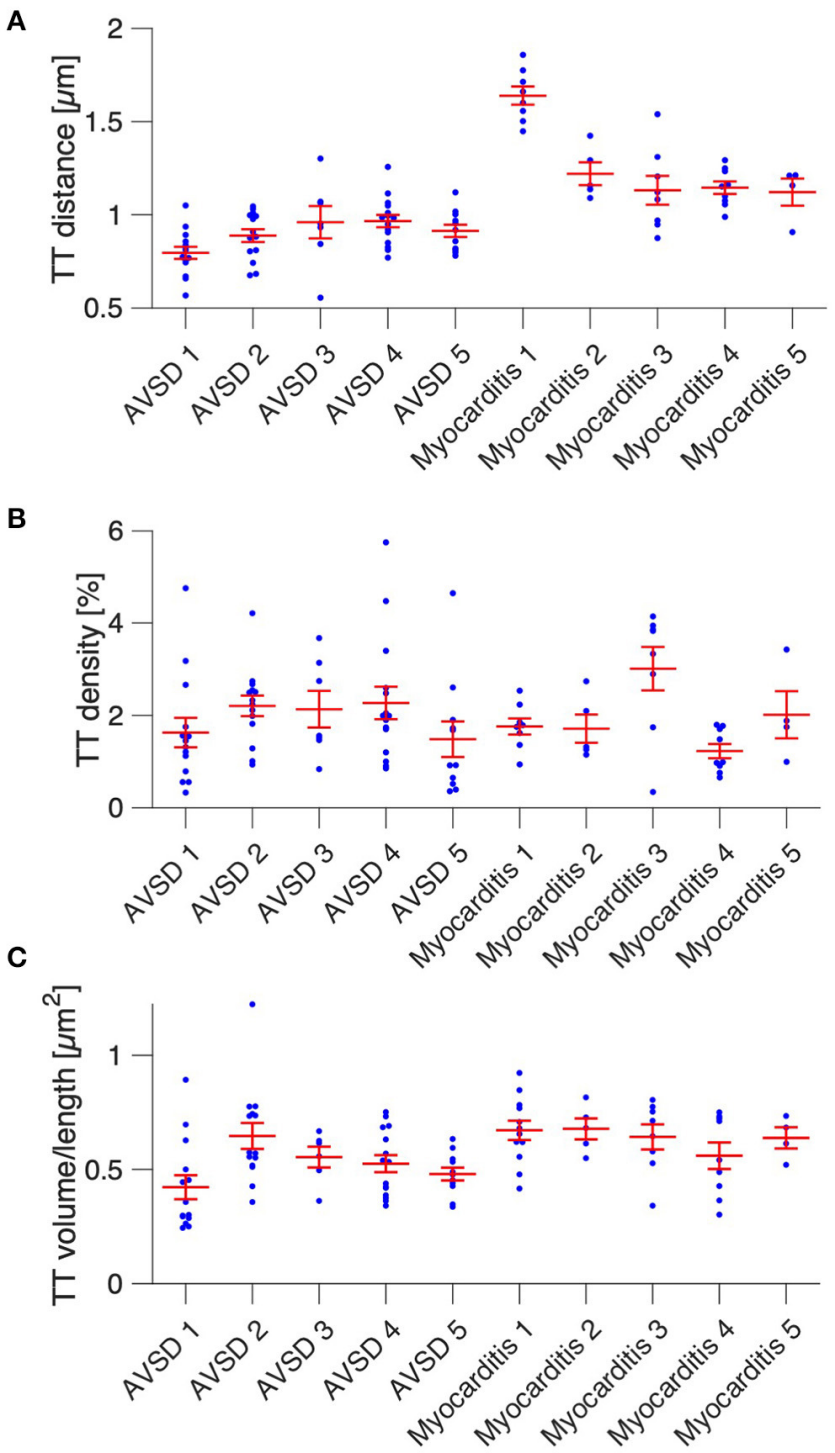
Cardiomyocyte t-system parameters grouped by patient. **(A)** Intracellular t-tubule (TT) distance of cells from five AVSD and five myocarditis patients (one sample per patient). **(B)** Cardiomyocyte TT density (TT volume divided by cell volume). **(C)** Mean cardiomyocyte TT volume-to-length ratio, a measure of TT enlargement. Number of analyzed cells: 13/14/6/15/11 (AVSD 1-5) and 8/5/8/9/4 (Myocarditis 1-5), respectively. One-way ANOVA (patient as categorical variable): *p* < 0.01 in **(A–C)**.

Next, we analyzed t-system remodeling quantitatively, comparing three different t-system measures of 61 cells from 5 patients with AVSD with 34 cells from 5 patients with myocarditis (Figure 2). As measures of t-system remodeling, we used intracellular t-tubule (TT) distance, calculated from distance maps of segmented cardiomyocytes (Figures 2A,B), TT volume density (TT volume normalized to cell volume) and TT volume-length ratio. The volume-length ratio is an estimate of mean t-tubule cross section area and an indicator of t-tubule dilation. An increase is associated with the presence of t-sheets (11, 24). TT distance was markedly increased in myocarditis when compared with AVSD (1.27 ± 0.05 μm vs. 0.90 ± 0.02 μm, respectively, *p* < 0.001, Figure 2C). The overall TT volume density, however, was not altered (1.94 ± 0.18% vs 1.95 ± 0.15%, respectively, *p* = 0.95). This may indicate fewer, but dilated t-tubules in myocarditis hearts. As expected from inspection of Figure 1, we found increased volume-length ratios in myocarditis (0.636 ± 0.024 μm^2^), when compared with AVSD cardiomyocytes (0.524 ± 0.023 μm^2^, *p* < 0.05). Signs of cellular hypertrophy were also present in myocarditis, because cell area, measured at the optical section with the largest area, was nearly doubled (1,260 ± 95 vs. 606 ± 48 μm^2^, *p* < 0.001). From one myocardits sample, isolated cardiomyocytes were analyzed instead of fixed tissue sections. We therefore tested if cell isolation might have affected the results in this sample (Supplementary Figure 1), but could not detect any differences in cell size or t-system measures. For this reason, the sample was included. An overview of the variability from sample to sample is depicted in Figures 3, 4. Figure 3 shows examples of confocal images from each patient. We observed t-sheets in cardiomyocytes from four out of five myocarditis samples (Figures 3F–J). At close inspection, sheet-like remodeled t-system components were also detected in cells from the AVSD sample diagnosed with hypertrophic cardiomyopathy (Figure 3B), but not in any other AVSD sample. Figure 4 displays the t-system measures from each sample, explaining the variation of data in Figure 2C and motivating the use of hierarichal statistical models. TT distance was elevated in all myocarditis samples, while TT density showed high variability. TT volume-length ratio was visibly increased in four out of five myocarditis samples, which confirms the observations in Figure 3. In agreement with these findings, one-way analysis of variance (ANOVA) indicated that the patients stem from different populations with respect to all three t-system measures (*p* < 0.01). In summary, increased TT distance and enlargement of t-tubules to t-sheets highly resemble the changes commonly observed in chronic heart failure in adult patients.

**Figure 5:**
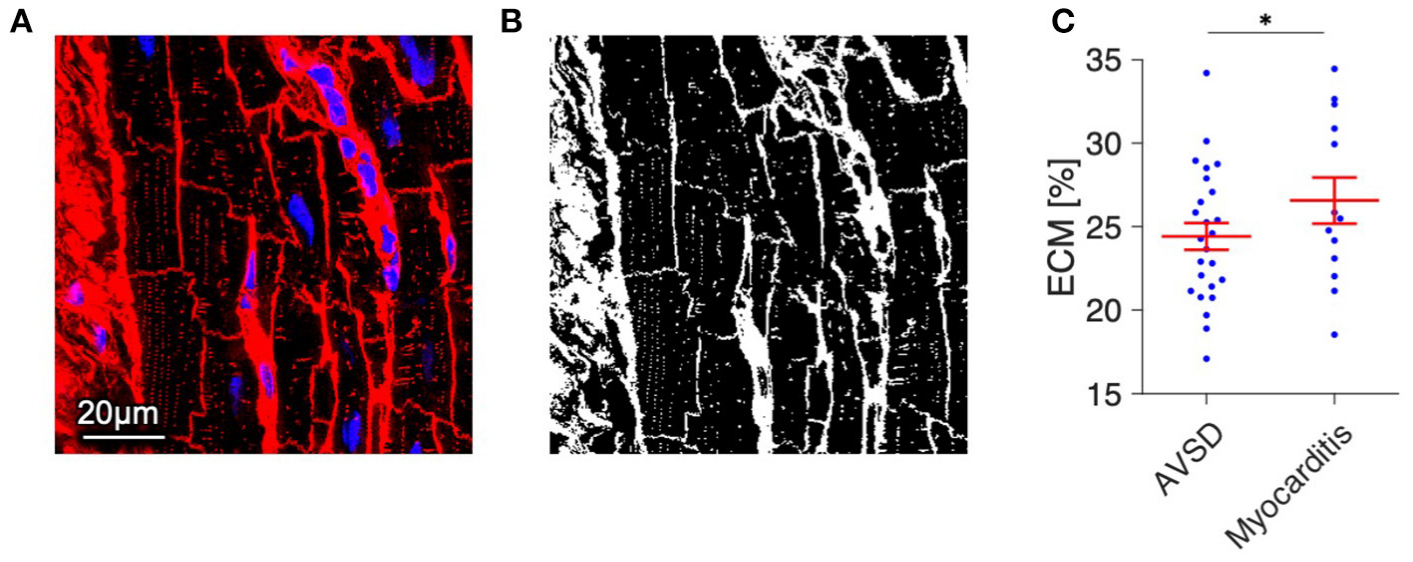
Quantification of extracellular matrix (ECM) as a measure of fibrosis in myocardial tissue from AVSD and Myocarditis patients. **(A)** Example of a confocal image from AVSD tissue stained with WGA (*red*) and DAPI (*blue*). **(B)** The resulting binary image after application of a histogram-based intensity-threshold was used to calculate the fraction of ECM. **(C)** ECM fraction of myocardial samples from 5 AVSD and 5 Myocarditis patients, obtained from 25 and 13 confocal image stacks, respectively. *p < 0.05, linear mixed-effects model with patient group (AVSD or Myocarditis) as predicting variable and intercept as random effect by patient (i.e., sample). Scale bar in **(A)** also applies to **(B)**.

Because t-system remodeling has been linked to fibrosis (13, 29), we tested for differences in the amount of extracellular matrix (Figure 5). The volume fraction of extracellular matrix was slightly increased in myocarditis tissue samples (26.6 ± 1.4%) when compared with AVSD samples (24.4 ± 0.8%, *p* < 0.05), but did not indicate severe fibrosis, as other studies investigating fibrosis near the infarct border zone by similar methods reported values of 30% to 40% (22, 30).

### No Evidence for T-System Recovery During Mechanical Circulatory Support

**Figure 6:**
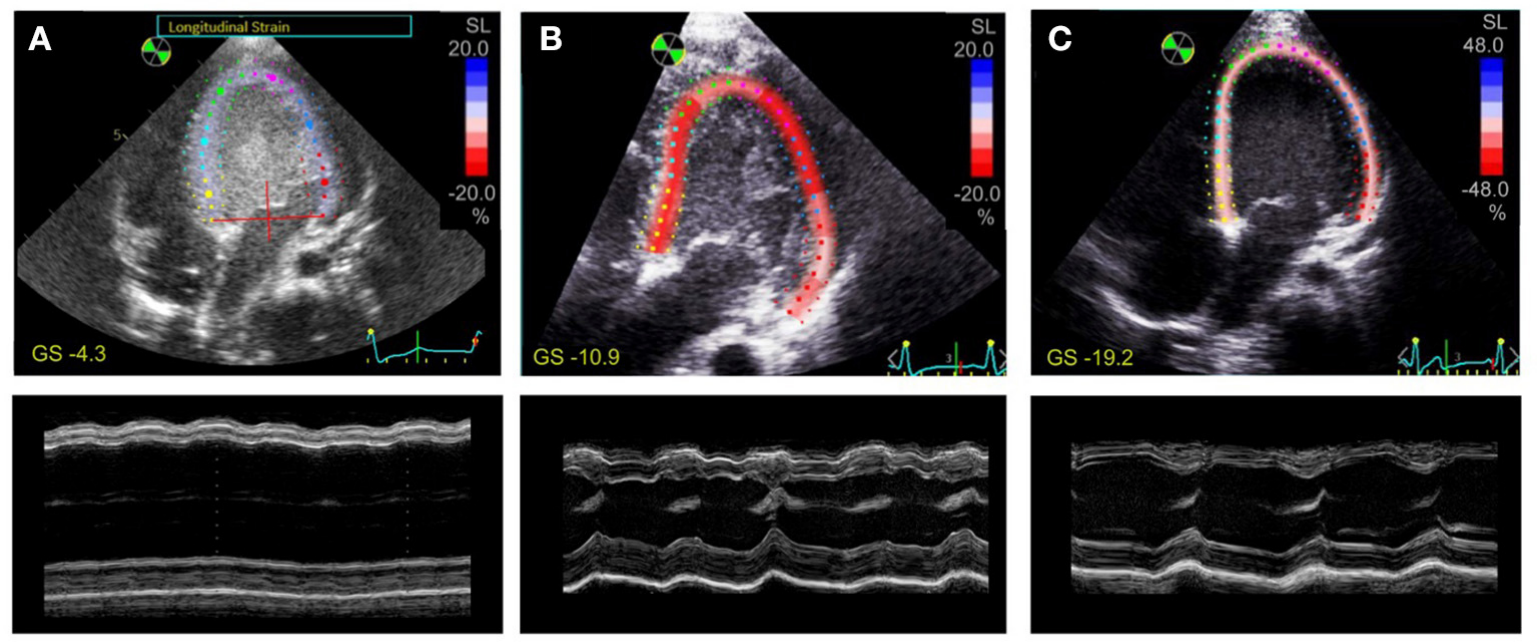
Speckle tracking and M-mode echocardiography of a 21-month old patient with fulminant viral myocarditis. Apical 4-chamber views with tracing of the endo- and epicardium and longitudinal strain (SL) of the left ventricle (*top*), and M-mode view (*bottom*) **(A)** 1 day before VAD implantation, **(B)** after 1 month of VAD therapy, **(C)** after 3 months of VAD therapy. Global strain (GS) is indicated in yellow. Note the different color scaling in **(C)**.

**Figure 7:**
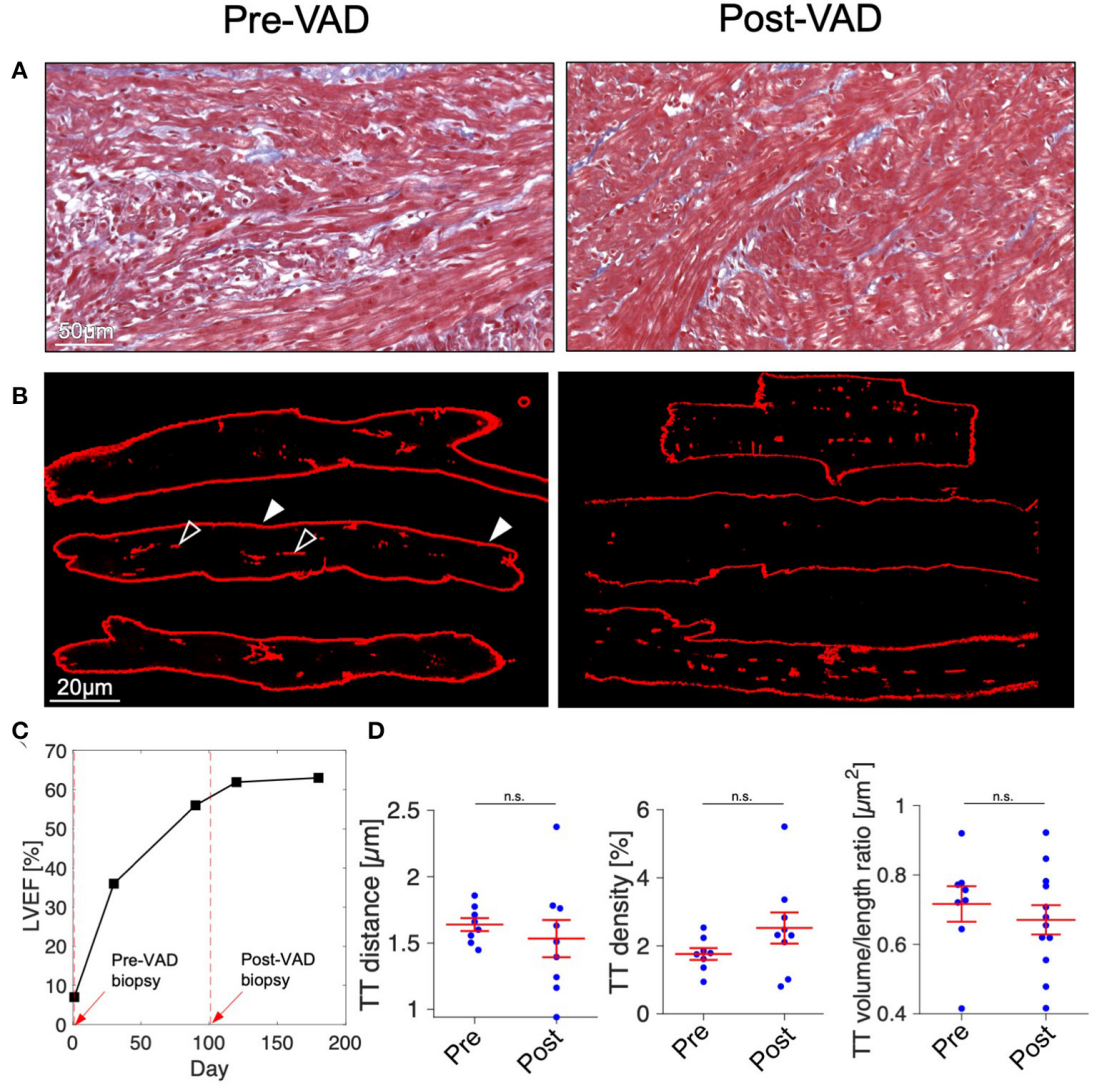
Histology and t-system quantification after functional cardiac recovery. **(A)** Masson's trichrome stain of myocardial tissue of a 21-month old myocarditis patient before VAD implantation (Pre-VAD) and at time of VAD explantation (Post-VAD) 3 months later. Cardiomyocytes appar red, collagen appears blue. **(B)** Pre- and Post-VAD confocal microscopic images of isolated and membrane-stained cardiomyocytes. In one example cell, filled arrow heads point to the surface membrane, empty arrow heads point to t-system components. **(C)** Overview of patient's cardiac function expressed as left-ventricular ejection fraction (LVEF). Dashed red lines and arrows indicate times of VAD implantation and explantation. **(D)** Cardiomyocyte t-tubule (TT) distance, TT density, and TT volume/length ratio (*n* = 8/9 cells for Pre- and Post-VAD, respectively). n.s., not significant (*p* > 0.05), unpaired two-tailed *t*-test.

Despite the rapid decline of cardiac function in fulminant viral myocarditis, affected pediatric hearts are able to recover (31). Mechanisms and structural correlates underlying recovery, however, remain only vaguely defined. To explore mechanisms of recovery, we investigated samples of a patient from which we had the opportunity of receiving a myocardial specimen also during VAD removal. VAD explantation was possible after significant clinical recovery. Figure 6 shows echocardiographic images of the 21-month old patient. Left-ventricular ejection fraction (LVEF) was 7% at the time of VAD implantation, and global longitudinal strain (GLS) was −4.3 (Figure 6A), both indicating a nearly complete lack of contraction. Cardiac function and strain recovered markedly during 3 months of mechanical circulatory support (Figures 6B,C), with LVEF reaching 56%, and global strain reaching −19.2 before explantation. To explore whether functional cardiac recovery was associated with structural recovery of the t-system and EC coupling junctions, we investigated these structures in more detail (Figure 7). Tissue morphology and collagen deposition of pre- and post-VAD biopsies were not visibly different (Figure 7A). Also, comparison of the t-system of isolated cardiomyocytes from pre- and post VAD did not provide evidence for t-system recovery because t-sheets were still widely present (Figure 7B), and TT distance and density were not significantly changed (Figure 7D). TT volume-length ratio remained at 0.67 ± 0.04 μm^2^ (pre-VAD: 0.72 ± 0.05 μm^2^), which exceeded markedly the value of AVSD samples (compare with Figure 2C) and was consistent with persisting t-sheets. In summary, these data suggest that, although cardiac function improved markedly, there was only marginal recovery of the overall t-system structure. Thus, t-system recovery might not explain the prominent functional recovery of the investigated heart during circulatory support.

### Remodeling and Recovery of EC Coupling Junctions

**Figure 8:**
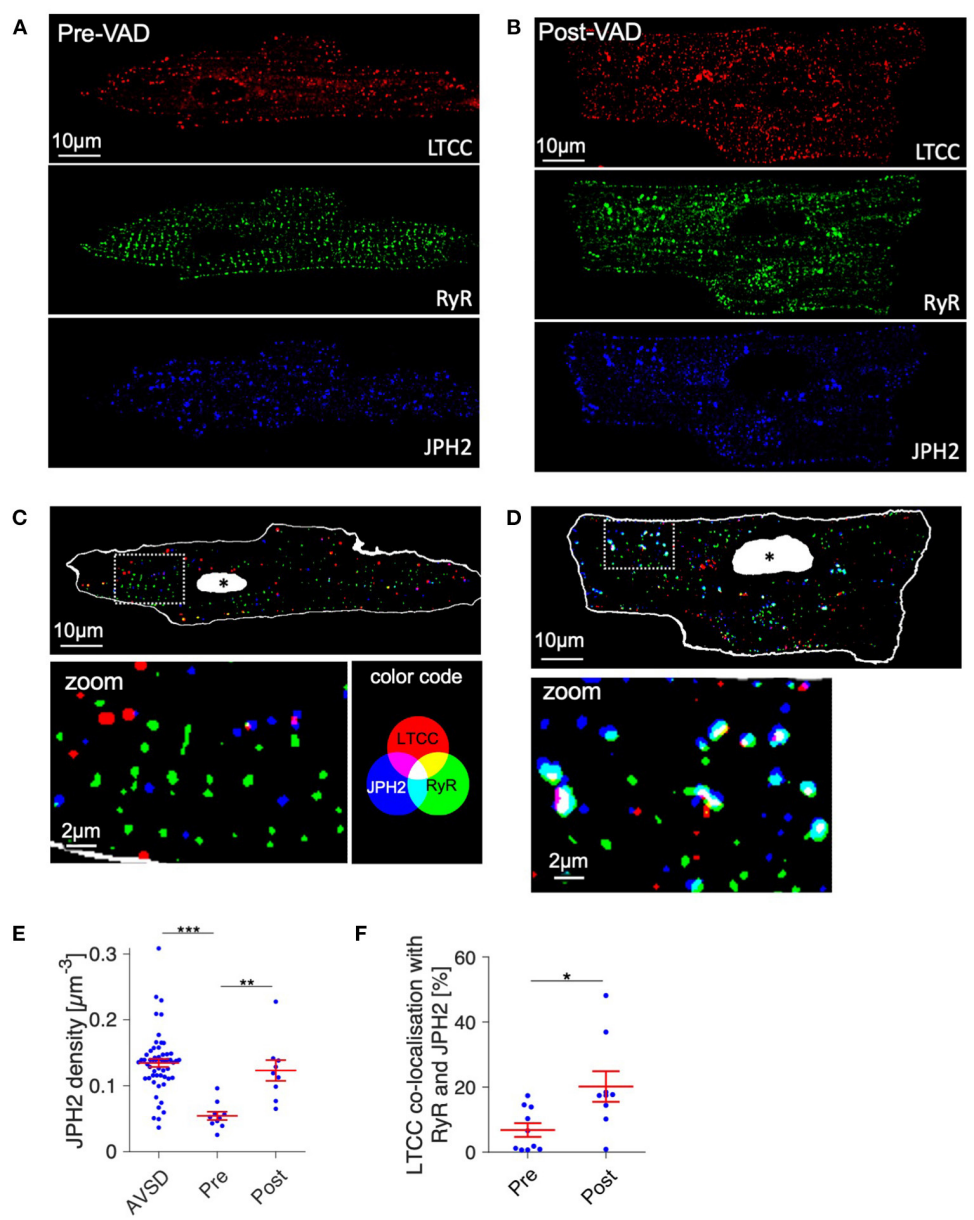
Structural integrity of excitation-contraction (EC) coupling junctions before and after VAD therapy of a myocarditis patient. **(A,B)** Raw confocal images of fixed isolated cardiomyocytes from pre- and post-VAD of the patient presented in Figures 6, 7, co-stained for LTCC (red), RyR (green), and JPH2 (blue) and with DAPI (not shown). **(C,D)** Overlay of binary images for the EC coupling proteins shown in **(A,B)**, with magnifications of boxed regions. The cell surface, obtained from autofluorescence, is shown white, nuclei are shown white with black asterisk. Co-localizations of LTCC, JPH2, and RyR appear cyan, magenta, yellow, or white (see color legend). **(E)** Cardiomyocyte JPH2 cluster density (JPH2 density) of AVSD as reference and the Pre- and Post-VAD sample. **(F)** Fraction of LTCC clusters that were co-localized with both RyR and JPH2, as a measure of intact EC coupling junctions (*n* = 10/9 cells for Pre/Post-VAD). *p < 0.05, **p < 0.01, ***p < 0.001, unpaired, two-tailed *t*-test with Holm–Bonferroni multiple-comparison correction.

To investigate whether other structures of EC coupling might be responsible for functional recovery we immunostained the junctional proteins junctophilin-2 (JPH2), L-type Ca^2+^ channels (LTCC), and cardiac ryanodine receptor (RyR) in myocytes obtained pre- and post-VAD and analyzed the integrity of EC coupling junctions by confocal microscopy (Figure 8). From raw confocal images shown in Figures 8A,B, the overall appearance of clusters seemed similar between pre- and post-VAD cells. However, inspection of Figures 8C,D revealed a larger number of LTCC clusters in co-localization with JPH2 and RyR in post-VAD myocytes, which was hardly visible in pre-VAD myocytes. This indicates a greater fraction of functional EC coupling junctions. Cluster analysis of the segmented confocal images (Figure 8E) showed extensive recovery of the JPH2 cluster density during VAD therapy, reaching values comparable to the AVSD control group (pre: 0.05 ± 0.01 μm^−3^, post: 0.12 ± 0.02 μm^−3^, *p* < 0.001 *n* = 10/9 cells, respectively; AVSD: 0.13 ± 0.05, *n* = 56 cells). Furthermore, cluster analysis confirmed a significantly higher co-localization of LTCC with JPH2 and RyR in post- than pre-VAD (pre: 6.8 ± 2.2%, post: 20.2 ± 5%, *p* < 0.05, Figure 8F). Because LTCC staining quality was insufficient in fixed tissue preparations, no co-localization analysis was performed in the AVSD group (see Supplementary Figure 2). In conclusion, the results indicate improved JPH2 densities and a higher density of intact EC coupling junctions at time of VAD explantation, suggesting a potential molecular basis for the observed functional cardiac recovery.

## Discussion

Acute myocarditis in young children frequently causes heart failure (15, 32), but the mechanisms leading to contractile dysfunction are poorly understood. Also, the determinants of sustained recovery are largely unclear. Here, we report severe t-system loss and abnormal t-tubule structure (t-sheets) in young children suffering from viral myocarditis. Such severe t-system remodeling is commonly observed in chronically failing adult hearts (7, 9), correlates with HF duration and may impair cardiac recovery (11), but has to our knowledge not been described in children. Thus, our results provide evidence that remodeling of the t-system is not restricted to chronic pathologies in adult patients, but can as well occur in young children and acute heart failure. We also shine light on recovery of EC coupling junctions related to improved cardiac function after mechanical circulatory support.

### T-System Remodeling in Children and Acute Heart Failure

Generally, little is known on the t-system in human infants and young children. One study reported a lack of t-tubules in newborns and an already dense t-system in 5- and 7-month old infants (33). This fits well to our finding that the t-system in the control group (age: 9 – 27 months) was dense and contained regularly-shaped t-tubules. It is therefore likely that in humans, as in other mammals, the t-system develops during early infancy (34–37) and that cardiac EC coupling relies on a dense t-system not only in adults, but also in children. Of note, this renders t-tubule loss a possible pathomechanism in pediatric heart disease. The pronounced loss of t-tubules in the children with viral myocarditis investigated here (Figure 2) may have contributed to the acute reduction of cardiac output and is in accordance with studies reporting diminished contractility after acute t-system loss in de-tubulated animal myocytes or myocardium (38, 39). Presuming that t-system alterations in the studied patients developed along with clinical symptoms, the sudden onset of cardiac dysfunction suggests that t-system deterioration in humans may arise in short periods of time, as observed in some animal models (40, 41). This would have important implications when considering that so far only very limited evidence exists for the possibility of structural t-system recovery (42, 43). Acute heart failure, for example due to myocarditis, could become chronic when involving t-system remodeling. Future studies could investigate this hypothesis by probing t-system remodeling in acute heart failure and subsequently after clinical recovery or chronification.

We also found markedly greater sizes of cardiomyocytes in the myocarditis group, which is in accordance with reports of transient hypertrophy in pediatric myocarditis (44) and a general association of myocarditis with hypertrophy (45). However, hypertrophy is not considered a classical symptom of myocarditis. Thus, our finding might result from investigating particularly severe cases which required VAD implantation.

### Inflammation as a Possible Trigger of T-System Remodeling

The triggers of t-system loss *in vivo*, especially in human hearts, remain elusive. Excessive strain and wall stress (46), mechanical load (47), fibrosis (13, 29), and dysregulation of several t-system associated proteins (48) have been suggested. We found only mildly increased amounts of extracellular matrix in myocarditis samples (Figure 5), rendering it unlikely that fibrosis was the sole trigger of t-system remodeling, because previous studies reported much higher degrees of fibrosis in association with t-system alterations (13, 29). Although increased mechanical load is present in fulminant myocarditis, we suggest the hypothesis that t-system loss is facilitated or triggered by inflammatory pathways. These are highly active in myocarditis (14, 49), but also in chronic heart failure (50) and after myocardial infarction (51), where t-system remodeling is regularly observed. Excessive activation of inflammatory signaling in cardiomyocytes, for instance through viral infection, could affect cellular processes required for t-system maintenance. Disturbed autophagy, for example, was reported to contribute to t-system loss in isolated cardiomyocytes (24), while viral proteases are able to degrade proteins of the autophagic machinery (18). Interestingly, it was recently shown that glucocorticoids prevent t-system loss in cardiomyocyte culture and that glucocorticoid receptor knockout causes t-system loss *in vivo* (24). As glucocorticoids exert anti-inflammatory effects, e.g., via NF-κB inhibition (52), and NF-κB inactivation has been shown to preserve calcium handling (53), it seems possible that downregulation of inflammatory pathways protects from t-system remodeling. However, these concepts require further investigation, and it should be noted that although treatment with glucocorticoids seems favorable especially in non-viral myocarditis (54), it may also have adverse effects (55).

### Structural and Functional Recovery

Analysis of the t-system and junctional proteins in the sample obtained after circulatory support and clinical recovery revealed only marginal recovery of the t-system. Although TT density was increased, TT distance and morphology (presence of t-sheets) were far from normal. The volume-length ratio, an indicator of t-tubule dilation to t-sheets (11), was still elevated and could explain why TT volume density reached nearly normal values, while TT distance—the presumably most relevant parameter for EC coupling (56)—remained increased. One should consider, however, that the biopsy at explantation was taken adjacent to the scar caused by VAD implantation and might therefore differ structurally from other regions of the ventricle. Although no major differences of collagen deposition between pre- and post-VAD biopsies were apparent (Figure 7A), overall fibrosis, altered strain patterns near the apex and microscale deposition of ECM could have impeded t-system recovery particularly at the biopsy site. Thus, the question remains open whether t-system recovery is generally possible.

Regarding the integrity of EC coupling junctions, however, cardiomyocytes showed clear improvements after mechanical circulatory support, which may have contributed to improvements in contractile function. JPH2 cluster density improved despite persistent t-system remodeling and reached levels of the control AVSD group. This confirms other studies reporting that JPH2 degradation and t-system remodeling are accompanied in cardiac disease (12, 57), but it also suggests that they may recover independently from each other. Because JPH2 is thought to improve coupling between LTCC and RyRs (1–4), it is likely that the integrity of EC coupling junctions ameliorated secondary to increased JPH2 expression, even though there was no t-system recovery (Figure 7). This would fit to observations of high plasticity of junctophilin-2 and ryanodine receptors (4) and to the observation that Ca^2+^ release is more rapid and more efficient in compact dyads, i.e., EC coupling junctions of high integrity (58). Investigating this idea in more detail and finding ways to induce reverse remodeling of EC coupling junctions could be promising targets of future studies.

### Limitations

The t-system was imaged in WGA-stained, fixed tissue sections in 9 out of 10 samples, while from the sample used for recovery analysis (Figure 6) isolated cells stained with Di-8-ANEPPS were imaged. Both WGA and Di-8-ANEPPS are widely used reliable markers of the t-system in humans and animals (7, 9, 11, 24, 37), but it has been reported that t-tubules and caveolae can decline quickly after cell isolation (59, 60) and that PFA fixation may alter tissue dimensions (61). To examine if these effects may have confounded t-system measures, we stained and imaged tissue sections and isolated myocytes from a human ventricular biopsy with both methods, but could not detect differences in t-system density or morphology (Supplementary Figure 1). In addition, statistical analysis of AVSD vs. myocarditis, excluding the isolated cardiomyocytes, still yielded significantly increased TT distance in myocarditis (*p* < 0.001).

Because AVSD hearts may have been subjected to increased load due to valve defects, it is possible that the t-system in these samples was not completely normal. However, considering that increased cardiac load has been associated with a loss rather than a gain of t-tubules (47), it is unlikely that the detected t-system loss in myocarditis resulted from a flawed control. We also acknowledge that samples were obtained from different cardiac regions in AVSD (LV outflow tract) and myocarditis hearts (LV apex), but although t-tubule remodeling may vary regionally, it seems to correlate with function rather than with specific regions of the heart (9). To substantiate the results regarding recovery of the t-system and EC coupling junctions, studies with a larger number of cases are required to enable statistical analysis. The case explored here shall generate new hypotheses and motivate studies in this field.

### Conclusions

The results from the presented study indicate that remodeling of EC coupling junctions and the t-system can occur in young children and may contribute to contractile dysfunction in acute viral myocarditis. Furthermore, this study provides strong evidence of severe t-tubule loss and remodeling to t-sheets in acute heart failure, challenging the idea that chronic heart failure or ischemic cardiomyopathy is required to elicit t-system remodeling in humans. Although t-system recovery remains elusive, recovery of EC coupling junctions may be possible and deserves further investigation. Moreover, we suggest to further explore the t-system as a prognostic biomarker (11, 62) not only in adults but also in pediatric heart disease.

## Data Availability Statement

The raw data supporting the conclusions of this article will be made available by the authors, without undue reservation.

## Ethics Statement

The studies involving human participants were reviewed and approved by Institutional Review Boards of the University of Tuebingen and the Friedrich-Alexander-University (FAU) Erlangen-Nuremberg. Written informed consent to participate in this study was provided by the participants' legal guardian/next of kin.

## Author Contributions

MA, SD, KK, TS, and TV contributed to the conception and design. RC, DF, and MS contributed to the data acquisition and experiments. MA, DF, and TS contributed to the data analysis. MA, DF, TS, and TV contributed to the interpretation of the data. DF and TS drafted the manuscript. All authors critically revised and approved the manuscript.

## Conflict of Interest

The authors declare that the research was conducted in the absence of any commercial or financial relationships that could be construed as a potential conflict of interest.
