# Supplementary figures and images for: Severe T-System Remodeling in Pediatric Viral Myocarditis
Source: Front Cardiovasc Med. 2021 Jan 18;7:624776. doi: 10.3389/fcvm.2020.624776 (PMC7848076; doi:10.3389/fcvm.2020.624776)

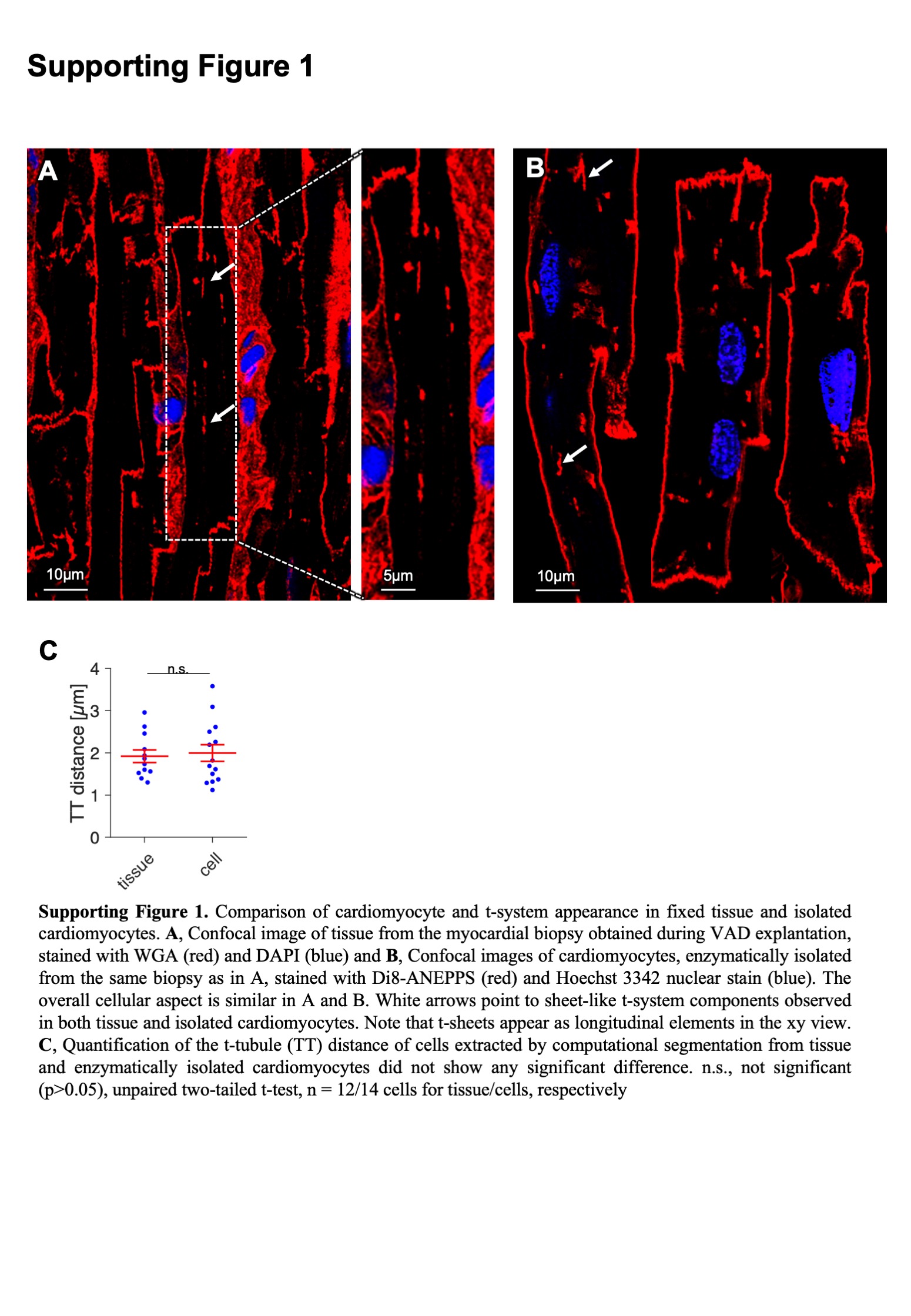

Supplement: Supplementary file 1 [file Image_1.JPEG]

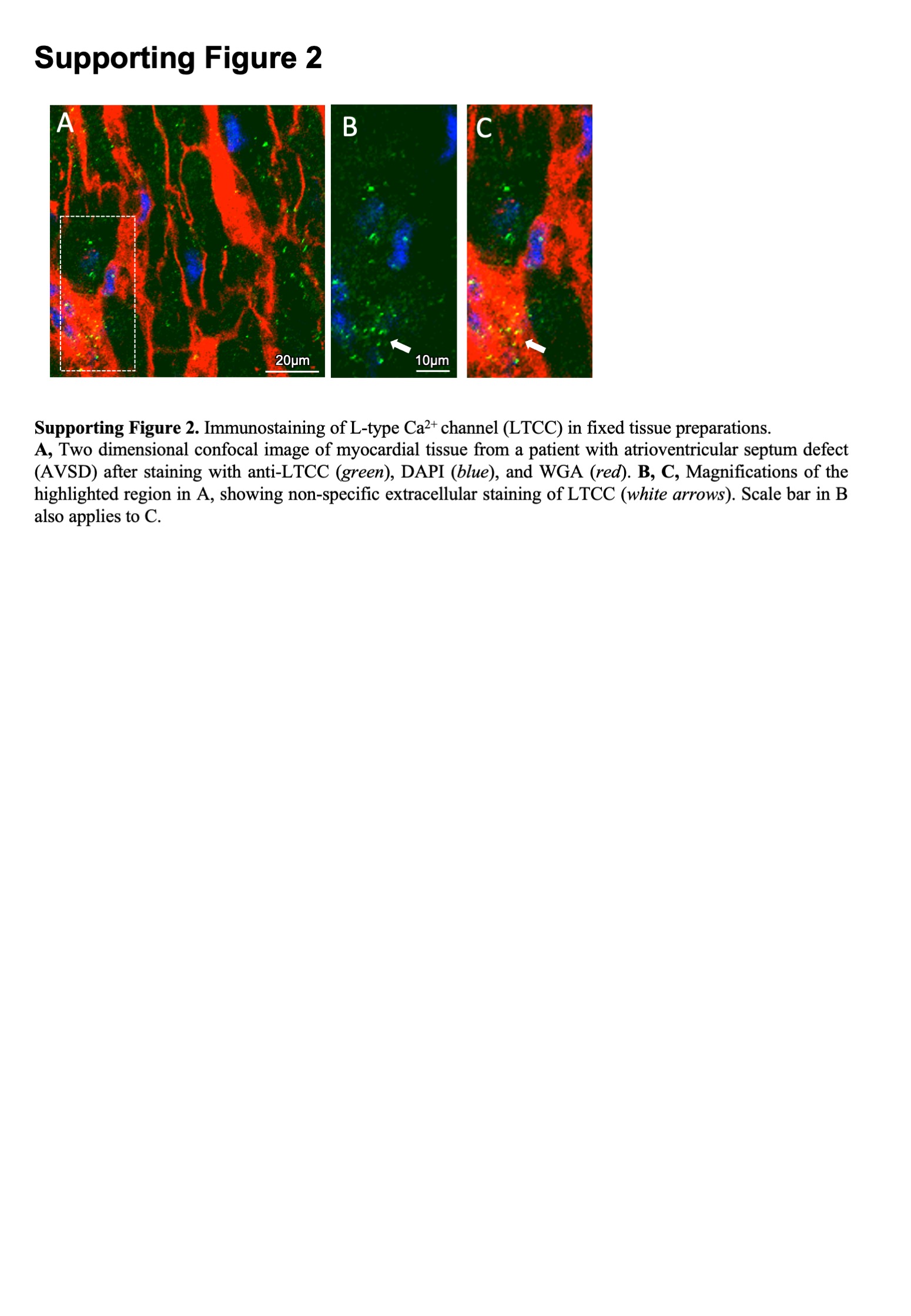

Supplement: Supplementary file 2 [file Image_2.JPEG]
